# Mouse Models That Enhanced Our Understanding of Adult T Cell Leukemia

**DOI:** 10.3389/fmicb.2018.00558

**Published:** 2018-03-28

**Authors:** Sara Moodad, Abdou Akkouche, Rita Hleihel, Nadine Darwiche, Marwan El-Sabban, Ali Bazarbachi, Hiba El Hajj

**Affiliations:** ^1^Department of Internal Medicine, Faculty of Medicine, American University of Beirut, Beirut, Lebanon; ^2^Department of Anatomy, Cell Biology and Physiological Sciences, Faculty of Medicine, American University of Beirut, Beirut, Lebanon; ^3^Department of Biochemistry and Molecular Genetics, Faculty of Medicine, American University of Beirut, Beirut, Lebanon; ^4^Department of Experimental Pathology, Immunology and Microbiology, Faculty of Medicine, American University of Beirut, Beirut, Lebanon

**Keywords:** adult T cell leukemia, HTLV-I, mouse models, Tax, HBZ

## Abstract

Adult T cell Leukemia (ATL) is an aggressive lymphoproliferative malignancy secondary to infection by the human T-cell leukemia virus type I (HTLV-I) and is associated with a dismal prognosis. ATL leukemogenesis remains enigmatic. In the era of precision medicine in oncology, mouse models offer one of the most efficient *in vivo* tools for the understanding of the disease biology and developing novel targeted therapies. This review provides an up-to-date and comprehensive account of mouse models developed in the context of ATL and HTLV-I infection. Murine ATL models include transgenic animals for the viral proteins Tax and HBZ, knock-outs for key cellular regulators, xenografts and humanized immune-deficient mice. The first two groups provide a key understanding of the role of viral and host genes in the development of ATL, as well as their relationship with the immunopathogenic processes. The third group represents a valuable platform to test new targeted therapies against ATL.

## Introduction

### Human T Cell Leukemia Virus

Human T-cell leukemia virus type I (HTLV-I) retrovirus belongs to the deltaretroviridae family of viruses (reviewed in Matsuoka and Jeang, 2007). It is the first described oncogenic retrovirus and is responsible for a spectrum of diseases, the most aggressive of which is Adult T Cell Leukemia (ATL) (Poiesz et al., 1980; Hinuma et al., 1981; Yoshida et al., 1982, reviewed in Watanabe, 2017; Zhang et al., 2017). Approximately 5–20 million people are infected with HTLV-I worldwide (reviewed in Gessain and Cassar, 2012). However, the highest prevalence is reported in endemic areas that include Japan, the Caribbean, South America, inter-tropical Africa, Pacific islands, some areas in the Middle East, and Romania (Nosaka et al., 2017, reviewed in Edlich et al., 2003; Gessain and Cassar, 2012). The genome of this virus encodes for classical structural proteins required for retroviral replication and a series of accessory and regulatory proteins including the viral transcriptional activator Tax (Lee et al., 1984, reviewed in Azran et al., 2004) and the HTLV-I bZIP factor gene (HBZ), a viral protein encoded from the 3′ long terminal repeat (LTR) in the complementary strand of the proviral genome (Gaudray et al., 2002, reviewed in Matsuoka and Jeang, 2011; Giam and Semmes, 2016; Ma et al., 2016). Both Tax and HBZ are linked to HTLV-I pathogenesis (reviewed in Boxus and Willems, 2009; Kannian and Green, 2010;Giam and Semmes, 2016).

### Adult T Cell Leukemia

Adult T cell leukemia is an aggressive hematological malignancy with very poor prognosis, high relapse rate, resistance to therapy, and a limited survival rate (Takatsuki et al., 1977; reviewed in Hermine et al., 1998; Bazarbachi et al., 2004, 2010; Goncalves et al., 2010; Marcais et al., 2013; Nasr et al., 2017; Watanabe, 2017). ATL develops in around 5% of infected carriers, following a long latency period exceeding 20 years (reviewed in Ishitsuka and Tamura, 2014; Bangham and Ratner, 2015).

Adult T cell leukemia is characterized by the presence of leukemic cells with atypical morphology and lobulated nucleus (Shimoyama et al., 1983). The majority of these cells are mature CD3^+^ CD4^+^ CD25^+^ CD7^-^ cells exhibiting an increased expression of the alpha chain of interleukin 2 receptor (IL-2R) but also an overexpression of Foxp3, a marker of T regulatory (T_reg_) cells (Waldmann et al., 1984; Shimoyama, 1991; Okayama et al., 1997; Karube et al., 2004).

### Tax as a Viral Oncoprotein

Tax is a 40 kDa viral transactivator protein that promotes viral transcription via the 5′-LTR. Tax functions as an oncogene resulting in leukemia (Grassmann et al., 1989; Tanaka et al., 1990). In rat fibroblasts, the expression of Tax is sufficient for induction of transformation and development of tumors (Grassmann et al., 1989, 1992; Tanaka et al., 1990). Tax trans-activates transcription by activating promoters implicated in cell proliferation, activation, and survival leading to accumulation of diverse genetic and epigenetic mutations, genetic instability, cell cycle checkpoint disruption, and damage of DNA repair mechanisms (reviewed in Marriott and Semmes, 2005; Kfoury et al., 2012; Bazarbachi, 2016; Watanabe, 2017). Tax binds critical transcription factors including the cAMP response element binding protein (Zhao and Giam, 1992; Brauweiler et al., 1995; Giebler et al., 1997), AP-1 (Fujii et al., 2000; Iwai et al., 2001), and serum response element (SRF) (Fujii et al., 1991, 1992). In addition, Tax inactivates tumor suppressor genes including p53 (Pise-Masison et al., 1998; Portis et al., 2001) and p16 (Suzuki et al., 1996), represses the expression of cyclin A, and antagonizes apoptosis through inhibiting apoptotic genes expression such as Bax and promoting anti-apoptotic ones including Bcl-xL and BFI-1 (Brauweiler et al., 1997; Nicot et al., 2000; Marriott and Semmes, 2005; Macaire et al., 2012).

Importantly, Tax activates the NF-κB pathway (Sun et al., 1994; Good and Sun, 1996; Mori et al., 1999; Hironaka et al., 2004), after binding the regulatory subunit of the IkappaB kinase (IKK) complex called nemo or IKK-γ (Harhaj and Sun, 1999; Kfoury et al., 2008; Wang et al., 2016) resulting in the activation of downstream effector genes (reviewed in Kfoury et al., 2005). Furthermore, Tax induced NF-κB activation is highly dependent on its post translational modifications namely ubiquitylation and sumoylation (reviewed in Kfoury et al., 2012). Tax also affects the expression of various micro RNAs (mi-RNA) including mi-RNA31 known to inhibit the expression of the NF-κB non-canonical pathway components (Yamagishi et al., 2012). Tax-induced mi-RNA31 downregulation occurs via a deregulation of polycomb proteins, leading to a consequent activation of NF-κB, and inhibition of apoptosis in ATL cells (Yamagishi et al., 2012). Moreover, Tax modulates the microenvironment and increases ATL cells’ invasion and extravasation through affecting gap junctions between endothelial cells and infected cells (El-Sabban et al., 2002; Bazarbachi et al., 2004).

Due to genetic/epigenetic alterations in the HTLV-I genome, including mutations and promoter methylation, most ATL cells lack detectable Tax expression (Takeda et al., 2004). Despite its undetectable levels in ATL patients (Matsuoka and Jeang, 2007), Tax is essential for ATL cells survival as its silencing results in cell death (Dassouki et al., 2015; Bazarbachi, 2016). Recent data revealed transient Tax expression in the form of Tax bursts (Tax expression switching on/off) occurring in a small fraction of ATL-derived or HTLV-I transformed cells (Mahgoub et al., 2018, reviewed in Bangham and Matsuoka, 2017). In a similar context, ATL epigenome was analyzed and an ATL-specific “epigenetic code” paramount for cell identity was deciphered (Fujikawa et al., 2016). In more details, Tax was shown to induce an epigenetic-dependent global alteration including increased polycomb complex 2 (PRC2) trimethylation at histone 3 (H3k27me3) resulting in cellular transformation, immortalization, and epigenome reprogramming, similar to that observed in ATL patients (Fujikawa et al., 2016). All these properties make Tax an ideal oncoprotein for *in vivo* investigation.

### HBZ Biology in ATL

HBZ is a nuclear protein encoded by the complementary strand of HTLV-I RNA genome (Larocca et al., 1989; Gaudray et al., 2002). Unlike Tax that is often undetected in ATL cells, *Hbz* gene undergoes no abortive mutations and the protein is expressed in all ATL patients and HTLV-I infected carriers (Fan et al., 2010; Kataoka et al., 2015; reviewed in Satou et al., 2006; Matsuoka and Jeang, 2011). HBZ was found to be a negative regulator of Tax-mediated viral transcription (Gaudray et al., 2002). This opposite expression pattern of the two proteins may indicate a possible differential role in HTLV-I pathogenesis and suggests HBZ as a candidate for a possible HTLV-I vaccine (Mahieux, 2015; Sugata et al., 2015). The mRNA of HBZ positively correlates with the proviral load of HTLV-I in carriers, and ATL patients (Saito et al., 2009). *In vitro*, HBZ promotes the proliferation of ATL cells but its suppression by short hairpin RNA (shRNA) results in modest inhibition of ATL cells proliferation (Satou et al., 2006; Arnold et al., 2008). HBZ affects several cellular pathways implicated in cellular proliferation such as NF-κB (Zhao et al., 2009; Panfil et al., 2016), AP-1 (Matsumoto et al., 2005), JunD (Thebault et al., 2004; Kuhlmann et al., 2007), c-Jun, JunB (Basbous et al., 2003), and CREB (Lemasson et al., 2007). In contrast to Tax which constitutively activates both canonical and non-canonical NF-κB pathways, HBZ was shown to inhibit the canonical pathway of NF-κB via proteasomal degradation of p65 while the non-canonical pathway was not affected (Zhao et al., 2009; Panfil et al., 2016).

### Animal Models in ATL

Due to the complexity of HTLV-I associated diseases and the enigmatic mechanisms dictating their occurrence, in particular in ATL, animal models have been instrumental in providing a platform for answering pivotal questions related to HTLV-I infection, disease progression, and importantly developing new effective therapeutic approaches (reviewed in El Hajj et al., 2012; Niewiesk, 2016). Among these models, rabbits, monkeys but also rats were useful to understand early HTLV-I viral infection and transmission as well as the induced host immune response against the virus (reviewed in El Hajj et al., 2012). More recently, transgenic *Drosophila* models expressing Tax in the compound eye and plasmatocytes were generated (Shirinian et al., 2015). However, mice remain by far one of the most efficient tools helping in understanding the biology of this affliction. Murine ATL models include transgenic animals for the viral proteins Tax and HBZ, xenografts inoculated with ATL cells (either cells lines or patient-derived cells) and humanized mouse models (reviewed in Panfil et al., 2013; Niewiesk, 2016). In this review, we attempt to provide an updated summary of these various mouse models, the key advances they offered in the understanding of HTLV-I infection, as well as their contribution to ATL research and drug development.

## Mouse Models of ATL

### Immunocompromised Mouse Models

Mice are relevant tools to study the molecular mechanisms of carcinogenesis and to develop new antitumor therapies. However, in immunocompetent mice, transplantation is often hindered by the functional host immune response resulting in low or no tumor engraftment. This problem was overcome after the discovery of the immunocompromised CB17 *scid*/*scid* (SCID) mouse model making a revolution in the cancer field. These mice harbor a spontaneous non-sense mutation in the *scid* gene, encoding for the protein kinase DNA activated catalytic polypeptide (Pkrdc), indispensable for efficient B and T lymphocytes recombination (Bosma et al., 1983). The loss of *Pkrdc* results in impaired adaptive immunity whereby B and T cells are both non-functional. Despite the lack of adaptive immunity, SCID mice retain a normal innate immunity in which macrophages, antigen-presenting cells, and natural killer (NK) cells carry normal functions (Bosma et al., 1983).

To further improve tumor engraftment, a non-obese diabetic (NOD/SCID) model exhibiting additional mutations resulting in further impairment of NK activity was generated (Shultz et al., 1995). This model was further immunosuppressed to generate the NOD/SCID β2-microglobulin^null^ mice in which the *β2-microglobulin* gene was deleted resulting in a complete abolishment of the NK cell activity (Koller and Smithies, 1989). Importantly, a NOD/SCID IL2-Rγ^-/-^ or NSG model was generated by deletion or truncation of the gamma chain of IL-2R (Ito et al., 2002), reviewed in (Ito et al., 2008). Therefore, in addition to all the abnormalities of their predecessors, NSG mice possess a defective production of IL-2, IL-4, IL-7, IL-9, IL-15, and IL-21 as well as a severe impairment of the dendritic cell (DC) and their capacity to produce interferon γ (IFN-γ) upon stimulation (Ito et al., 2002; Ishikawa et al., 2005). For further immunosuppression, the Rag2^-/-^γc^-/-^ model was established. These mice have a deletion of the Recombination Activating Genes (*RAG2*), impairing the production of both T and B cells and NK cell-mediated immunity in murine hosts. Moreover, because the rag proteins are not involved in DNA repair, *RAG2*-deleted mice do no show the leakiness or radio-sensitivity observed in SCID mice (Traggiai et al., 2004; reviewed in Chicha et al., 2005). Because of these properties, these mice allowed to study human hematopoiesis (Hiramatsu et al., 2003; Ishikawa et al., 2005; reviewed in Pearson et al., 2008).

### ATL Development in Xenograft Mouse Models

The use of xenograft mice has provided invaluable information pertaining to the tumorigenic and proliferative potential of ATL (Ohsugi et al., 1994). Under this section, we will provide an overview of most tested immunocompromised animals injected with HTLV-I-transformed or ATL-derived cell lines or those injected with patient-derived ATL cells. Initial xenograft studies investigating ATL development and progression were performed in the SCID mouse model (Ishihara et al., 1992; Feuer et al., 1993; Kondo et al., 1993). Later studies reported the use of NOD/SCID and SCID/Beige mice for injection of transformed or immortalized cell lines (Liu et al., 2002). More recently, an HBZ xenograft mouse model was generated by transplantation of retrovirally transduced T cells with Bcl-xL, AKT, and HBZ (Kasugai et al., 2016). Only mice groups transplanted with T cells triply transduced with plasmids encoding for the three proteins generated tumors, highlighting the need of key components from different cellular pathways along with the viral HBZ for cellular transformation (Kasugai et al., 2016) (**Table 1**).

**Table 1:** Summary of the contribution of mouse models to ATL biology.

Model	Contribution to ATL biology	Reference
**Xenografts**		
Xenograft mouse models injected with HTLV-I transformed, ATL-derived cell lines or ATL patients derived cells	Represented a platform for targeted drug development. Provided a potential tool to test therapeutic agents:– Targeting the constitutively active NF-κB pathway. – Specifically targeting leukemic cells. Allowed a better understanding of the immune response against HTLV-I. Was instrumental for studying early stages of primary HTLV-I infection and subsequent clonal proliferation. Allowed the assessment of early steps of HTLV-I infection, proviral load, and clonal proliferation.	Zimmerman et al., 2011;Ishitsuka et al., 2012;Masaki et al., 2013;[Bibr B135][Bibr B125];Zhang et al., 2003, 2005;Maeda et al., 2010, [Bibr B93][Bibr B61];Feuer et al., 1995;Stewart et al., 1996;Uchiyama, 1996;[Bibr B89][Bibr B104][Bibr B149]
**Humanized**		
HTLV-I infected human CD34^+^ in Rag2^-/-^ gamma c^-/-^ mice models	Allowed the understanding of early infection stages. Confirmed the *in vivo* correlation of Tax and NF-κB activation upon expansion of CD4^+^CD25^+^ malignant cells.	Villaudy et al., 2011
HTLV-I infected human CD133^+^ in NSG	Generated a human adaptive immune system in immunodeficient mice. Was the closest model to recapitulate the *in vivo* ATL development.Assessed the initiated immune system against the virus and clonal selection.	Tezuka et al., 2014
***Tax* Transgenics**LTR-*Tax*-Tg	Established Tax as an oncoprotein and HTLV-I as transforming virus resulting in mesenchymal tumors and neurofibroma.	Hinrichs et al., 1987;Nerenberg et al., 1987
	Showed that Tax expression in oxidative fibers resulted in HTLV-I associated myopathies.	Nerenberg and Wiley, 1989
	Showed that Tax induced autoimmune like Sjogren like syndrome.	Green et al., 1989
	Showed that Tax induced skeletal abnormalities and fragile bones similar to ATL patients.	Ruddle et al., 1993
	Showed that Tax is arthrogenic and induced ankylotic arthropathy.	Habu et al., 1999
	Showed that Immune system activation contributes to ATL pathogenesis in infected carriers.	Swaims et al., 2010
Double transgenic -β*gal*-Tg modelLTR-*Tax* HTLV-I LTR	Unveiled tissues supporting tax-mediated transcriptional transactivation. Provided a model system to study the mechanism of gene regulation by Tax.	Bieberich et al., 1993
huGMZB*Tax*GMZ-*Tax*-TgGranzyme promoter	First model to generate leukemia (LGL), tumor infiltration, and splenomegaly partly resembling ATL.	Grossman et al., 1995
	Showed that Tax functionally inactivates P53 contributing to late stage tumor progression.	Portis et al., 2001
	Showed that innate immune system, specifically IFN-γ, is crucial for ATL development.	Mitra-Kaushik et al., 2004
	Revealed malignant hypercalcemia and osteolytic bone lesions resembling human ATL.	Gao et al., 2005
	Showed that Tax expression *in vivo* induced constitutive activation of HTLV-I.	Bernal-Mizrachi et al., 2006
	Revealed that Tax activation of lymphocytes recruits, activates, and transforms NK/T-cells.	Rauch et al., 2009b
CD3- *Tax* TgCD3-epsilon promoter/enhancer	Model failed to develop leukemia. Tax expression closely associated with apoptosis *in vivo*.	Hall et al., 1998
tTA/*Tax* miceBi-transgenic doxycycline inducible model, EmuSR alpha promoter-enhancer	Revealed ATL-like cutaneous lesions and splenomegaly via HTLV-I activation. Showed that Tax or HTLV-I suppression resolves cutaneous symptoms.	Kwon et al., 2005
lck-*Tax*-tg modelLck-proximal promoter	Showed diffuse large cell lymphoma after prolonged latency. Model exhibits acute ATL like symptoms and HTLV-I activation.	Hasegawa et al., 2006
	Provided a candidate ATL stem cells of CD38^-^/CD71^-^/CD117^+^ phenotype and decreased expression of Tax, Notch, BMI1 were isolated.	Yamazaki et al., 2009
lck-*Tax*-tg modelLck-Distal promoter	Showed leukemia of mature CD4^+^ cells resembling mature CD4^+^ ATL cells	Ohsugi et al., 2007b
***HBZ* transgenic***HBZ*-Tg modelCD4-specific promoter/enhancer/silencer	Showed systemic inflammation and later lymphoma in 30% of mice upon aging.	Satou et al., 2011
	Increased effector/memory CD4^+^ cells and functionally impaired CD4^+^ Foxp3^+^ T_reg_ cells.	Yamamoto-Taguchi et al., 2013
	Showed that HBZ promotes a pro-inflammatory phenotype via labile Foxp3 expression.	Sugata et al., 2012
	Showed that HBZ suppresses Th1 cytokines and impairs cell-mediated immunity.	Kuribayashi et al., 2016
	*HBZ*-Tg mice exhibit ATL stem cells of c-kit^+^/CD4^-^/CD8^-^ phenotype.	
**Double Transgenic *Tax/HBZ****HBZ/Tax* modelCD4 promoter/enhancer	This model failed to generate ATL-like leukemia. Skin lesions, T-cell lymphoma, and splenomegaly with increased CD4^+^ memory and Foxp3^+^T_reg_ cells.	Zhao et al., 2014

#### Xenograft Mouse Models as Platforms for ATL Targeted Drug Development

Given its resistance to therapy and its high relapse rate, ATL remains an aggressive disease with an unfavorable prognosis (reviewed in Bazarbachi et al., 2011; Nasr et al., 2017). Despite remarkable progress in ATL therapies with the use of antiviral therapy and allogeneic stem cell transplantation (Bazarbachi et al., 2010, 2014), most patients relapse highlighting the necessity of novel therapeutic approaches (**Table 2**).

**Table 2:** ATL mouse models as platform for ATL targeted therapy.

Model	Drug	Drug target	ATL therapy	Reference
Xenograft model	Bay 11-7082	NF-κB	Prevented tumor growth and infiltration.	Dewan et al., 2003
	Bortezomib	NF-κB	Prevented tumor growth in an ED SCID.	Satou et al., 2004
	DHMEQ	NF-κB	Prolonged survival and Prevented tumor growth.	Ohsugi et al., 2007a
	9-aminoacridine and Campath-1H	NF-κB and CD25	Prolonged survival and induced P53-mediated apoptosis.	Ju et al., 2014
	Compound E, Bortezomib, and Romidepsin	γ-secretase, NF-κB, and HDAC	Assessed interaction between Notch-1 and HTLV-I pathways, combination exhibited synergy supporting clinical trials.	Yu et al., 2015
	AR-42	HDAC	Prolonged survival.	Zimmerman et al., 2011
	Campath-1H and HAT or MEDI 507	CD2 and CD25	Targeting different CD25 epitopes exhibited synergy.	Zhang et al., 2006
	Flavopiridol and HAT	cyclin-dependent kinase and CD25	Synergy enhancing antitumor effect and survival.	Chen et al., 2009
	HAT, MAT, and 7G7B6	CD25	Inhibited tumor growth.	Maeda et al., 2010
	7G7/B6 and daclizumab	CD25	Presented osteoponin-integrin interaction as novel therapeutic target for ATL.	Maeda et al., 2015
	Daclizumab and Depsipeptide	CD25 and HDAC inhibition	Improved survival and attenuated tumor infiltration and viral production.	Ikebe et al., 2013
	A20 ShRNA	A20, ubiquitin-editingEnzyme	Decreased tumor growth and revealed a novel role for ubiquitin-editing enzymes in ATL development.	Saitoh et al., 2016
	ABT-737	Bcl-2 and Bcl-xL inhibition	Inhibited tumor growth.	Ishitsuka et al., 2012
	Adoptive patient-autologous Tax-CTL	ATL cells	Decreased tumor infiltration and enhanced survival *in vivo.*	Masaki et al., 2013
Humanized model	Tinofovir and Azidothymidine	Reverse Transcriptase inhibition	Prophylactic potential by blocking primary infection *in vivo.*	Miyazato et al., 2006
	TARC-PE38	CCR-4	CCR4 is a potential ATL target.	Hiyoshi et al., 2015
Transgenic model	Arsenic/IFN	NF-κB, Tax, LIC	Cured ATL via LIC elimination.	El Hajj et al., 2010
	ST1926	NF-κB, Tax, LIC	Highlights retinoids as promising therapies by enhancing survival and decreasing tumor infiltration.	El Hajj et al., 2014

*DHMEQ, dehydroxymethylepoxyquinomicin; HDAC, histone deacetylase; HAT, humanized anti-Tac; MAT, murin anti-Tac; arsenic, arsenic Trioxide; IFN, interferon-alpha; LIC, leukemia initiating cells.*

##### Targeting the NF-κB pathway in ATL therapy

Adult T cell leukemia development entails the deregulation of multiple cellular pathways, including the constitutive activation of the NF-κB pathway (reviewed in Kfoury et al., 2005), making this pathway an attractive therapeutic target against ATL. Accordingly, a wide array of NF-κB inhibitors including specific (Bay 11-7082), and non-specific inhibitors (such as bortezomib and Dehydroxymethylepoxyquinomicin DHMEQ), were tested in ATL xenograft mouse models.

Bay 11-7082, specifically inhibiting the NF-κB DNA binding activity, prevented primary tumor growth and leukemic organ infiltration in NSG mice xenografted with HTLV-I infected cell lines (Dewan et al., 2003). Bortezomib prevented tumor growth in an ED, ATL-derived T cell line, xenografted SCID model (Satou et al., 2004). DHMEQ, known to inhibit the activation of NF-κB by preventing the nuclear translocation of its active subunit p65 (Ohsugi et al., 2006), showed a significantly prolonged survival and prevented tumor growth in an ATL NSG xenograft mouse model (Ohsugi et al., 2007a).

In a similar context, a small molecule, 9-aminoacridine (9AA) selectively induced *in vitro* ATL cell death through inhibition of the NF-κB pathway and induction of p53 responsive genes (Ju et al., 2014). The efficacy of 9AA alone or in combination with Campath-1H (a monoclonal antibody directed against CD52) was assessed using a xenograft NOD/SCID model, inoculated with MET-1 cells. MET-1 are activated T cells that express CD2, CD3, CD4, CD25, CD122, and CD52. An enhanced survival of tumor bearing mice was reported upon treatment with both compounds as compared to either compound alone. This involved the induction of p53, PARP cleavage, and apoptosis of splenic cells from leukemia-bearing mice (Ju et al., 2014).

Finally, and in order to assess the physical and functional interaction between Notch-1 signaling (increased in some ATL patients) and NF-κB activation, the therapeutic efficacy of the γ-secretase inhibitor compound E along with bortezomib and romidepsin, a histone deacetylase inhibitor (HDACI), was explored in an NSG model injected with MT-1 cells (Yu et al., 2015). Each of the three reagents alone or in double combination inhibited tumor growth as monitored by tumor size, the level of tumor markers in the serum, and significantly prolonged the survival of tumor-bearing animals (Yu et al., 2015).

At the cellular and molecular levels, and to assess the *in vivo* implication of NF-κB in ATL pathogenesis, Nitta et al. (2008) investigated ATL development in a defective NF-κB setting. In this study, alymphoplasia *(aly/aly)* mice bearing an NF-κB inducing kinase (NIK) mutation were used. These mice harbored defects in lymphoid organs development and severe deficiencies in both humoral and cell-mediated immunity. In contrary to BALB/c and C57BL/6J control mice, *aly/aly* animals inoculated with HTLV-I producing MT-2 cells, did not maintain the provirus and antibodies against HTLV-I were not detected suggesting that NIK is required for the initial proliferation and maintenance of HTLV-I infected cells in mice (Nitta et al., 2008).

##### Targeted ATL drug development other than NF-κB inhibition

One of the suggested approaches in ATL treatment is virotherapy. Oncolytic virotherapy is a relatively new approach in cancer treatment, utilizing replication-competent viruses that selectively target cancer cells while sparing the normal healthy ones (Russell et al., 2012). MET-1 NOD/SCID model was used to evaluate the potential of measles-virus-virotherapy in treating ATL. Measles virus treatment of tumor cells lacking type I interferon (IFN-α) secretion was shown to be more efficient both *in vitro* and *in vivo* as compared to other tumor cells (Parrula et al., 2011). Using the same animal model, Zimmerman et al. (2011) investigated the therapeutic effect of AR-42, an HDACI, in alleviating HTLV-I-associated lymphoid malignancies. A dietary formulation of AR-42 was shown to prolong survival of ATL engrafted mice as compared to controls.

Non-obese diabetic/SCID xenograft mice were also used to test ABT-737, a small molecule inhibitor of Bcl-2 and Bcl-xL, whereby ABT-737 resulted in a significant inhibition of the tumor growth *in vivo* (Ishitsuka et al., 2012).

In another study, Ikebe et al. (2013) investigated the efficacy of 17-DMAG, an HSP90 inhibitor, as a therapeutic agent against ATL. Oral administration of 17-DMAG dramatically attenuated the aggressive infiltration of multiple organs in an ATL xenograft mouse model. It also inhibited the *de novo* viral production and improved the overall survival of ATL mice (Ikebe et al., 2013).

More recently, an unrecognized role of ubiquitin-editing enzyme, A20, in the survival of HTLV-I-infected cells was unveiled in a SCID model. In brief, the ATL-derived HuT-102 cell line was first transduced with A20 shRNA and then inoculated into SCID mice (Saitoh et al., 2016). Depletion of A20 induced apoptosis and affected the *in vivo* growth of HTLV-I infected cells, highlighting the importance of ubiquitin in ATL development.

##### Monoclonal antibodies as an ATL therapy in xenograft mouse models

The expression of specific cell surface markers on ATL cells implanted into mice, renders them an excellent model for testing the pre-clinical therapeutic potential of monoclonal antibodies. In this context, and given that ATL cells express CD2 and CD25 among other surface markers, the effect of monoclonal antibodies targeted against these two surface markers was performed in NOD/SCID mice intraperitoneally injected with MET-1 cells (Zhang et al., 2003). Furthermore, the effect of campath-1H (anti CD52), either alone or in combination with a humanized anti-Tac (HAT) or MEDI 507, a monoclonal antibody directed against CD2, was investigated. Campath-1H led to a striking prolongation of the survival of MET-1 ATL-bearing mice. This survival was significantly longer than that of the group receiving HAT. Moreover, the study revealed the anti-leukemic mechanism of action of Campath-1H which involves FcR-gamma-containing receptors (e.g., FcRgamma-III) present on polymorphonuclear leukocytes and macrophages, known to normally mediate antibody-dependent cellular cytotoxicity (ADCC) and/or trigger cross-linking induced apoptosis (Zhang et al., 2003). The same group explored the use of flavopiridol, a cyclin-dependent kinase inhibitor, alone or in combination with HAT. HAT/flavopiridol combination resulted in a prolonged survival and dramatic enhancement of the antitumor effect in MET-1 NOD/SCID mice as compared to the control group (Zhang et al., 2005).

A MET-1 NOD/SCID mouse model was also used to investigate the anti-leukemic effects of specific antibodies targeting IL-2R (Phillips et al., 2000) whereby HAT, murine anti-Tac (MAT), and 7G7/B6, all of which targeting IL-2Rα, significantly delayed leukemia progression resulting in enhanced survival (Phillips et al., 2000). To decipher the mechanism of action of these antibodies, comparison between treated-NOD/SCID and NSG mice was carried out (Zhang et al., 2004). In contrast to what was seen in NOD/SCID mice, treatment of NSG mice did not affect leukemia growth nor improved mice survival highlighting that the immune system difference, specifically polymorphonuclear cells, plays a crucial role in leukemia elimination by anti-IL-2R antibodies (Zhang et al., 2004).

Another promising therapeutic target is CC chemoreceptor 4 (CCR4). CCR4 is a chemokine receptor expressed by tumor cells in about 90% of ATL patients (Ishida et al., 2003). Several studies have investigated the effect of monoclonal Anti-CCR4 antibodies *in vivo* as potential therapies in ATL (Yano et al., 2008; Ito et al., 2009; Ishii et al., 2010; Hiyoshi et al., 2015). Using an ATL SCID xenograft mouse model, Yano et al. tried to augment the ADCC effect induced by defucosylated chimeric Anti-CCR4 IgG1 monoclonal antibody KM2760 *via* addition of granulocyte colony stimulating factor (G-CSF). Addition of G-CSF resulted in a more robust antitumor effect as compared to KM2670 alone (Yano et al., 2008). Using the same model, another monoclonal anti-CCR4 antibody KW-0761 was investigated (Ishii et al., 2010). KW-0761 also presented promising antitumor activity where tumor volume was significantly decreased (Ishii et al., 2010).

Anti-CD25 antibodies such as 7G7/B6 and daclizumab, directed against different epitopes of CD25 were also tested either alone or in combination (Zhang et al., 2006). Overall, 91% of the mice receiving the combination survived showing the promising synergistic effect of these two antibodies, especially when compared to the single antibody treatment (Zhang et al., 2006). In a similar study and using the same model, daclizumab was investigated in combination with an HDAI, depsipeptide, and showed an enhanced antitumor effect and survival of the leukemia-bearing mice, compared with those in the depsipeptide or daclizumab alone groups (Chen et al., 2009).

Given that some ATL cells express CD30 on their surface, the therapeutic effect of two anti-CD30 monoclonal antibodies was also investigated. SGN-30, a chimeric anti-CD30 mAb, and SGN-35, a monomethyl auristatin E-conjugated anti-CD30 mAb, were used. Maeda et al. (2010) treated NOD/SCID mice subcutaneously engrafted with HTLV-I-infected cells and reported a significant inhibition of the tumor growth upon treatment with either antibodies (Maeda et al., 2010).

In another context, the therapeutic efficacy of adoptive patient-autologous Tax-specific cytotoxic T cells (Tax-CTL) was assessed in NSG mice bearing primary ATL cells from three patients. Tax-CTL treatment resulted in a significant decrease of ATL cell infiltration into blood, spleen, and liver as well as a significant prolonged survival time in ATL NSG mice that received cells from two out of three patients (Masaki et al., 2013).

More recently, the NSG mice were used to investigate the physiological roles of osteopontin (OPN)-integrin interaction in ATL pathogenesis *in vivo*. ATL cell lines inoculated into NSG mice resulted in an increased OPN plasma levels. Treatment of these mice with anti-OPN mAbs inhibited not only tumor growth but also tumor invasion and metastasis suggesting a pivotal role of OPN in these processes (Maeda et al., 2015).

Altogether, the above studies highlight the importance of xenograft mice models in targeting and understanding ATL.

### Humanized Mouse Models of ATL

Despite the importance of ATL xenograft mouse models, these models present with the limitation of being injected with ATL-derived or HTLV-I-transformed cell lines that were maintained for years in culture. This entails the potential changes in their genetic drift and the attenuation in their *in vivo* potential. Moreover, ATL xenograft mouse models do not provide answers to how HTLV-I induces ATL at early steps and how it maintains leukemogenesis (reviewed in Niewiesk, 2016). In this context, humanized mouse models were generated (reviewed in Panfil et al., 2013; Duc Dodon, 2014; Niewiesk, 2016) (**Table 1**).

#### Humanized Mouse Models: A Transformation in ATL Biology and Immune Responses

The lack of an adaptive and/or innate immune system is advantageous for HTLV-I replication and tumor engraftment. Injecting CD34^+^ hematopoietic stem cells into NSG mice resulted in generation of human lymphocytes. The first humanized ATL model was developed by Miyazato et al. (2006) in which human peripheral blood mononuclear cell (PBMC) were first injected in NSG mice to establish a human-like setting followed by inoculation of HTLV-I virus producing MT-2 cells. MT-2 cells ensured cell-to-cell transmission required for HTLV-I infection. In this model, proviral load was increased in both CD4^+^ and CD8^+^ cells (Miyazato et al., 2006).

In a similar study, PBMC from HTLV-I infected carriers were injected in NSG mice to establish HTLV-I infection (Takajo et al., 2007). Despite the different methodology, both studies resulted in mice harboring human infected cells, which later undergo clonal proliferation (Miyazato et al., 2006; Takajo et al., 2007).

Another humanized model was developed by Villaudy et al. (2011) whereby CD34^+^ human umbilical stem cells (HUSC) were intra-hepatically inoculated into newborn BALB/c/Rag22/2IL-2Rgc2/2 (also known as *BRG* mice) mice generating human lymphocytes. These lymphocytes were later infected by intraperitoneal injection of irradiated HTLV-producing MT-2 cell line. This study reported an alteration in T cell development alongside an increase in the proviral load and expansion of CD4^+^CD25^+^ cells. Mice also developed ATL- like splenomegaly as well as lymphoma (Villaudy et al., 2011).

In a different study, intra-bone marrow injection (IBMI) of cord blood CD133^+^ stem cells intratibialy into sublethally irradiated NSG mice was performed (Tezuka et al., 2014). This study is based on the idea that CD133^+^ cells are believed to be the ancestral of CD34^+^ in hematopoiesis and carry the potential to differentiate to any hematopoietic cells including lymphocytes (Tezuka et al., 2014; reviewed in Duc Dodon, 2014). One month following CD133^+^ inoculation, human CD45^+^ leukocytes were found to completely reconstitute the murine bone marrow. Later, human B and T lymphocytes were detected and a balanced B/T lymphocyte ratio was attained and remained stable for up to 8 months (Tezuka et al., 2014). After establishing a human immune system in this model, HTLV-I infection involved the injection of sublethally irradiated MT-2 cells known to produce HTLV-I. Shortly after infection, an increase in CD4^+^ cells was reported where CD4^+^ CD25^+^ clones gradually dominated indicating clonal selection. ATL like features including splenomegaly, hepatomegaly with ATL infiltration, and ATL-like “flower cells” were also reported (Tezuka et al., 2014). Screening of cytokines profiles demonstrated an initial elevation in the levels of IL-6, IL-8, IL-10, IL-12, IL- 13, IFN-γ, and TNF-α. In addition, granulocyte-macrophage colony-stimulating factor (GM-CSF) and chemokine (C-C motif) ligand 4 were also increased, clearly referring to an initiated immune response against the virus (Tezuka et al., 2014). One of the main findings of this study was the generation of a functional adaptive immune response in a humanized mouse model, summarized by detection of anti-HTLV-I antibodies as well as Tax-specific cytotoxic T cells (CTL). These CTL inversely correlated with proviral load of infected cells (Tezuka et al., 2014). Thus Tezuka’s model was by far the closest one in recapitulating the development of ATL *in vivo*. While most of the other humanized models developed lymphoma and/or thymoma, Tezuka’s model exclusively developed leukemia. This may be due to the fact that infection was carried out after a humanized immune system may be fully developed.

Another humanized ATL model was generated by Nakamura et al. (2015) where PBMC from ATL patients were injected into NOD/SCID/Jak3-null mice (NOJ mice). In Brief, the model involved subcutaneous injection of the ATL S1T cell line into mice, followed by subcutaneous, intraperitoneal, or intravenous transplantation of primary ATL cells. ATL cells successfully infiltrated various organs and could transplant into secondary mice establishing NOJ mice as successful models for primary ATL xenotransplantation (Nakamura et al., 2015).

#### Humanized Models as Platforms for ATL Therapy

After the revolution that humanized mouse models generated in the ATL field, they provided a strong platform for testing anti-ATL therapies (**Table 2**). Two antiviral reverse transcriptase inhibitors, Tenofovir and Azidothymidine, were tested in the first humanized ATL model developed by Miyazato et al. (2006). After the uncertain role of these agents in halting HTLV-I infection, their prophylactic potential was investigated. Both agents were reported to block primary infection in these mice (Miyazato et al., 2006).

Moreover, humanized models proved instrumental in investigating the anti-tumor potential of anti-CCR4 antibodies. NSG mice inoculated with primary ATL cells and autologous immune cells belonging to the same patient were used to assess the antitumor efficacy of KM2760 antibody (Ito et al., 2009). KM2760 decreased the number of ATL cells in blood, spleen, and liver, as well as ATL lesions and organ infiltration, and lowered IL-2R concentration in serum (Ito et al., 2009). Using a humanized mouse model similar to that developed by Villaudy et al. (2011), the efficacy of TARC-PE38 targeting CCR4 was investigated (Hiyoshi et al., 2015). TARC-PE38 is a complex of thymus and activation-regulated chemokine (TARC), CCR4 ligand, fused to a truncated *Pseudomonas aeruginosa* exotoxin A (PE38). TARC-PE38 efficiently killed HTLV-I-infected cell lines and shrank HTLV-I-associated solid tumors size. Moreover, TARC-PE38 markedly inhibited the proliferation of HTLV-I-infected human CD4^+^CD25^+^ or CD4^+^CD25^+^CCR4^+^ cells and reduced the proviral loads in PBMC obtained from both patients and asymptomatic carriers (Hiyoshi et al., 2015).

Using the NOJ model generated by Nakamura et al. (2015) the antitumor effect of pyrrolidine dithiocarbamate (PDTC), an antioxidant agent, was tested in ATL and showed that PDTC significantly enhanced the survival of these mice (Nakamura et al., 2015).

### Transgenic Mouse Models of HTLV-I

To decipher the oncogenic potential role of HTLV-I proteins *in vivo*, transgenic mice overexpressing the viral oncoproteins Tax or HBZ were generated. So far several *Tax* transgenic models expressing Tax under different promoters and two *HBZ* models were developed. Despite exhibiting many features of ATL, none of these models could exactly recapitulate HTLV-I-associated ATL. For instance, and as opposed to HTLV-I infection where HBZ- and Tax-specific CTLs and antibodies are generated, transgenics for both oncoproteins lack a host induced immune response against these viral proteins. the importance of transgenic models in the ATL context lies in confirming the oncogenic functions of HTLV-I proteins, Tax and HBZ, *in vivo* and in disclosing various host pathways manipulated by these proteins ultimately leading to tumor generation (**Table 1**).

#### Tax Transgenic Models Develop ATL-Like Leukemia/Lymphoma

##### Tax transgenic mice

The generation of *Tax* transgenic models relied on the earliest discoveries of the *in vitro* Tax oncogenic potential, from independent laboratories (Grassmann et al., 1989; Tanaka et al., 1990). To investigate the *in vivo* role of Tax in leukemogenesis, several mouse models have been generated (reviewed in Ohsugi, 2013; Niewiesk, 2016). Depending on the used promoter, Tax expression resulted in various tumors (Ohsugi, 2013). Except for models utilizing the lymphocyte-specific protein tyrosine kinase p56 (lck) or granzyme promoters, most of the models could not generate leukemia/lymphoma and rather resulted in less typical HTLV-I tumors and manifestations (Ohsugi, 2013; Panfil et al., 2013; Niewiesk, 2016). Nerenberg et al. (1987) established the first *Tax* transgenic mouse where *Tax* gene was under the control of the natural HTLV-I promoter, the LTR promoter [Tg (HIV-*tat*) 6-2Gja] (Nerenberg et al., 1987). Referred to initially as tat protein, Tax exhibited tissue-specific expression and LTR-*Tax* mice mainly developed mesenchymal tumors in the nose, ear, mouth, tail, as well as the foot. Despite not recapitulating human ATL, this research was novel in establishing Tax as an oncoprotein and HTLV-I as a transforming virus *in vivo* (Nerenberg et al., 1987). Beside mesenchymal tumors, LTR-*Tax* mice developed tumors at other multiple sites resembling neurofibroma making this model useful for studying neurofibromatosis (Hinrichs et al., 1987). Apart from tumors, mice were shown to develop myopathies similar to those associated with HTLV-I, which are due to the atrophy/degeneration of oxidative muscle fibers (Nerenberg and Wiley, 1989). Using the same model, Habu et al. (1999) reported high incidence of inflammatory polyarthropathy resembling arthritis. Profound skeletal alterations resulting in fragile bones with high turnover rate were also reported (Ruddle et al., 1993). Salivary and lacrimal glands showed exocrinopathy and lesions resembling Sjogren’s syndrome due to the attack by immune cells (Green et al., 1989).

Afterwards, a bi-transgenic mouse was generated by crossing LTR-*Tax* mice with LTR-β *gal* mice (β-galactosidase) to better visualize the organ involvement in ATL (Bieberich et al., 1993). Tax acts on the LTR resulting in an increased β-gal expression; and this enzyme was detected in specific tissues including bones, muscles, exocrine glands, as well as mesenchymal tumors (Benvenisty et al., 1992; Bieberich et al., 1993). Using this same model, Swaims et al. (2010) assessed the interaction with the host immune system and demonstrated that the activation of infected CD4^+^ T cells may induce Tax expression and thus may contribute to ATL pathogenesis in infected carriers. In addition, infected CD4^+^ cells harboring Tax exhibited changes in the expression of surface markers and resulted in changes in CD4^+^ subtype specifications (Swaims et al., 2010, reviewed in Kress et al., 2011). Despite not developing leukemia/lymphoma, the LTR-*Tax* model provided a clear-cut evidence that Tax expression is solely sufficient for tumor induction establishing Tax as an oncoprotein *in vivo*.

In another attempt, a *Tax* transgenic model of C57BL/6TgN mice (huGMZB*Tax*) was developed where *Tax* expression was controlled by the granzyme B promoter (Grossman et al., 1995). This promoter restricted the expression of Tax to the T cell compartment specifically CD4^+^ and CD8^+^, NK, and lymphokine activated killer cells. In this model, mice developed large granular lymphocytic leukemia (LGL), neutrophil dominated inflammation, as well as tumors on the ears, tail, and leg (Grossman et al., 1995). Mice also developed symptoms resembling human ATL such as high white blood cell count, splenomegaly, neutrophilia, and lymphadenopathy. LGL cells disseminated to distant organs such as lungs, bone marrow, and liver (Grossman et al., 1995). Despite not fully recapitulating the human disease, this model was instrumental in showing that Tax expression in the lymphocytes is sufficient to cause leukemia. Later, Gao et al. (2005) demonstrated that these mice also exhibited malignant hypercalcemia and symptoms associated with metastasis such as osteolytic bone lesions which again resemble the disease in ATL patients (Gao et al., 2005). Activated NK and T cells from this model demonstrated Tax-mediated constitutive activation of NF-κB in both its canonical and non-canonical pathways (Bernal-Mizrachi et al., 2006). Using the same model, the role of p53 inactivation in Tax-induced tumor development was assessed and showed that the p53 apoptotic pathway was functionally inactivated (Portis et al., 2001). P53 mutations in tumors were also detected but were associated with secondary organ infiltration. In the same context, mating of these *Tax*-transgenic mice with P53-deficient mice did not accelerate the initial tumor development (Portis et al., 2001). However, it significantly increased disease progression and mortality in *p53* heterozygous mice. This suggested that Tax functionally inactivates p53 which contributes to late stage tumor progression rather than initial tumor formation (Portis et al., 2001).

In a different study, the role of the innate immune system and inflammation in ATL development was assessed. The *Tax*-granzyme model was mated with an *IFN-*γ knock-out model. The resulting mice exhibited enhanced tumorigenesis with accelerated lesions development (Mitra-Kaushik et al., 2004). Using the same model, Rauch et al. generated *Tax*-LUC double transgenic mouse model whereby luciferase bioluminescent imaging techniques allowed to track tumor engraftment *in vivo* (Rauch et al., 2009a,b). The onset of peripheral subcutaneous tumors was preceded by the formation of microscopic intra-epithelial lesions. This suggests that Tax activates lymphocytes, which then recruit NK/T-cells to be activated and transformed (Rauch et al., 2009b). Another study from the same group suggested that in *Tax*-LUC model, lymphoma development is promoted by an inflammatory stimulus whereby T cell activation via T cell receptor (TCR) was shown to promote/exacerbate tumorigenesis (Rauch et al., 2009a). Afterwards, the same *Tax*-LUC model was utilized to investigate the role of IL-15 in spontaneous lymphoma development (Rauch et al., 2014). IL15^-/-^ *Tax*-LUC mice were generated and resulted in an aggressive lymphoma development and accelerated mortality, suggesting that IL-15 contributes to the antitumor immunity in ATL. Knocking out IL-15 also resulted in elevation of IL-1α and IL-1α driven cytokines (Rauch et al., 2014). Treatment with anti-IL-1α antibodies resulted in decreased tumor growth. This study suggested IL-15 and IL-1α as potential therapeutic options in ATL.

Hall et al. (1998) established another *Tax* transgenic model where *Tax* gene was under the control of *CD3-epsilon* promoter-enhancer sequences. These mice, of C57/CBA origins, developed a variety of tumors including salivary and mammary adenomas as well as mesenchymal tumors, specifically at wound sites, yet failed to develop leukemia (Hall et al., 1998).

In an attempt to target Tax expression to the leukocyte compartment, a bi-transgenic doxycycline inducible model [Tg (*EmuSR-tTa*) 83Bop] was generated (Kwon et al., 2005). This conditional “tet off” *Tax* transgenic model targeted both wild-type Tax and Tax mutants that selectively compromise NF-κB or CREB pathways to the leukocyte compartment. Wild type *Tax* transgenic mice developed a lethal cutaneous disease with skin lesions infiltrated by CD3^+^CD4^+^MHC-2^+^ T cells, similar to those seen in ATL patients. Mice also developed systemic lymphadenopathy and splenomegaly. Moreover, inflammatory cytokines including TNF-α, IL-6, IL1 α/β, IFN-γ were induced (Kwon et al., 2005). Of note, suppression of Tax by doxycycline administration resulted in disappearance of skin lesions directly linking Tax to this dermal pathogenesis (Kwon et al., 2005).

To further restrict Tax expression to the thymus compartment, being the site of maturation of T cells, the two *Lck* promoters, distal and proximal, were used. The proximal promoter drives gene expression in thymocytes while the distal one restricts gene expression specifically to mature T lymphocytes (Hasegawa et al., 2006; Ohsugi et al., 2007b; reviewed in Ohsugi, 2013). Hasegawa et al. (2006) generated a *Tax* transgenic model using the *lck* proximal promoter [C57BL/6-Tg (Lck-HTLV-I *Tax*)]. As compared to previous transgenic models, Hasegawa’s model was the closest to recapitulate ATL. In this model, mice developed CD4^-^CD8^-^CD44^+^CD25^+^ diffuse large cell lymphoma and leukemia after a prolonged latency period of around 18 months resembling the latency period required in humans to generate tumors (Hasegawa et al., 2006). Interestingly, mice exhibited clinical and histological resemblance to acute ATL seen in patients, such as characteristic “flower cells” in blood smears, lymphadenopathy, splenomegaly with extensive infiltration by lymphomatous cells, as well as distant organ infiltration of bone marrow, liver, kidney, lung, skin, and meninges. Infiltrating T cells were of malignant phenotype with increased expression of CD25^+^ cell surface marker (Hasegawa et al., 2006). In addition, mice exhibited further common features with ATL patients such as significant leukocytosis, hypercalcemia, elevated LDH, and constitutive NF-κB activation (Hasegawa et al., 2006). A major difference between Hasegawa’s mice and human ATL is in the phenotype of leukemic cells; mice exhibited immature CD4^+^ cells while human ATL involves leukemia generally of mature CD4^+^ cells (reviewed in Ohsugi, 2013). Since the development of leukemia required a long time, splenocytes derived from this *Tax* transgenic model were intraperitoneally injected in SCID mice. This model recapitulated most of the ATL phenotypes, similar to the transgenic model and to ATL patients, in less than 1 month (Hasegawa et al., 2006; El Hajj et al., 2010). Moreover, this model was the first to be used for the isolation of candidate ATL stem cells of CD38^-^/CD71^-^/CD117^+^ phenotype (Hasegawa et al., 2006; Yamazaki et al., 2009). These stem cells exhibited decreased levels of Notch, Tax, and BMI-1 expression all of which indicate their early hematopoietic cell origin (Yamazaki et al., 2009).

The distal promoter of *Lck* was also used to develop *Tax* transgenic mice (Ohsugi et al., 2007b). The expression of Tax under the distal promoter resulted in leukemia of mature CD4^+^ cells unlike the immature ones obtained in the Hasegawa et al generated *Tax* transgenic model (Ohsugi et al., 2007b).

##### Tax transgenic models for ATL-targeted therapy

Despite the improvements in prolonging survival of the leukemic subtypes of ATL upon using zidovudine and IFN-α (IFN) (Bazarbachi et al., 2010), most patients relapse underlying the urgent need for novel therapies and targets. Different drugs and drug combinations were investigated, of which a very promising treatment is the combination of arsenic trioxide (arsenic) and IFN-α (**Table 2**). *In vitro* studies involving ATL cell lines and primary patients’ leukemic cells showed the efficacy of the arsenic/IFN combination in triggering Tax degradation by the proteasome resulting in cell cycle arrest and apoptosis (Bazarbachi et al., 1999; El-Sabban et al., 2000; Nasr et al., 2003). To assess the efficacy of this combination *in vivo*, El Hajj et al. used spleen cells derived from the murine *Tax* transgenic ATL model (Hasegawa et al., 2006) and injected them into SCID mice (El Hajj et al., 2010). Approximately 1 month after transplantation, mice recapitulated ATL manifestations (Hasegawa et al., 2006; El Hajj et al., 2010). Strikingly, arsenic/IFN cured ATL in these mice. Although this combination did not rapidly decrease the tumor bulk, as ATL cells only underwent modest cell cycle arrest and apoptosis, the curative efficacy of arsenic/IFN occurred through clearance of leukemia initiating cells (LIC). Briefly, using the serial transplantation method, ATL cells derived from primary mice treated with the arsenic/IFN combination resulted in lower leukemia transplantation ability in untreated secondary mice and no transplantation in untreated tertiary mice (El Hajj et al., 2010). Addition of the proteasome inhibitor bortezomib to arsenic/IFN treatment of primary mice reversed all the observed phenotypes and led to normal ATL development in serial transplantation experiments, demonstrating that Tax degradation is the critical step for LIC exhaustion which further highlighted the oncogenic addiction of ATL cells to Tax (El Hajj et al., 2010). Later, Kchour et al. (2009) have investigated the effect of the triple combination of zidovudine, arsenic, and IFN, and translated the promising pre-clinical results to patients. Interestingly, the triple drug combination of zidovudine/arsenic/IFN resulted in 70% complete remission rate and 100% overall response rate in chronic ATL patients, as well as an unpreceded prolonged survival in some patients, strongly suggesting a similar mechanism of LIC eradication in patients (Kchour et al., 2009).

Using the same *in vivo* model, the preclinical efficacy of a synthetic retinoid ST1926 was investigated (El Hajj et al., 2014) (**Table 2**). Oral treatment of ST1926 induced massive apoptosis, prolonged survival and decreased tumor infiltration, leukocytosis, and splenomegaly as compared to untreated group of animals. This study highlights the potential of synthetic retinoids as a promising therapy for ATL (El Hajj et al., 2014). It remains to be determined whether ST1926 treatment alone targets LIC in ATL.

#### HBZ Transgenic Models Generate T-Cell Lymphoma but Not Leukemia

##### HBZ transgenic models

The first *in vivo HBZ* transgenic mouse model (*HBZ*-Tg mice) was generated by Satou et al. (2006), where the *HBZ* gene was expressed under the control of a murine *CD4*-specific promoter/enhancer/silencer (Satou et al., 2006). Restricted HBZ expression to CD4^+^ T cells resulted in systemic inflammation and development of T cell lymphoma in only 30% of mice after a long latency period. *HBZ*-Tg spontaneously developed systemic dermatitis, alveolitis, and later lymphoma upon aging (Satou et al., 2011). At the cellular level, HBZ increased the generation and proliferation of Foxp3^+^ T cells as well as the transcription of Foxp3 mRNA. *HBZ*-Tg mice exhibited an increase in the number of functionally impaired CD4^+^ Foxp3^+^ T_reg_ cells as well as effector/memory CD4^+^ T cells (Satou et al., 2011). Recently, this model was used to investigate whether the proliferation of CD4^+^ T cells is increased *in vivo*. Allergic encephalomyelitis was experimentally induced by immunization with myelin oligodendrocyte glycoprotein (MOG)/complete Freund’s adjuvant. Disease severity was not increased but the number of CD4^+^ T cells was increased only in the immunized *HBZ*-Tg mice suggesting that HBZ-expressing T cells have higher susceptibility to immune stimulation *in vivo* (Kinosada et al., 2017). Using the same model, Yamamoto-Taguchi et al. (2013) showed that HBZ promotes inflammation through labile Foxp3 expression; T_reg_ cells induced by HBZ have unstable Foxp3 expression and tend to convert to Foxp3^-^T cells producing IFN-γ. This HBZ-induced pro-inflammatory phenotype of CD4^+^ T cells was suggested to be involved in the HTLV-I-associated pathogenesis and inflammation (Yamamoto-Taguchi et al., 2013). Later, Mitagami et al. (2015) closely investigated HBZ-induced inflammation and revealed that in *HBZ*-Tg mice, inflammation severity significantly correlate with lymphoma development. The study suggested a link between HBZ inflammation and oncogenesis in CD4^+^ T cells (Mitagami et al., 2015).

In another context, HBZ expression was found to impair the cell-mediated immunity of HBZ-Tg via suppression of Th-1 cytokine production (Sugata et al., 2012). Despite being of low immunogenicity, anti-HBZ antibodies can be detected in patient’s serum. Sugata et al. (2012) have utilized the *HBZ*-Tg mouse model to investigate the possibility of generation of HBZ-targeted HTLV-I vaccine. Accordingly, splenocytes from HBZ mice, called HT-48, were inoculated into immunodeficient mice generating an ATL model. C57BL/6 mice immunized by a recombinant vaccinia virus-based HBZ vaccine generated HBZ-specific CD4 and CD8 T-cell response. Afterwards, inoculation of anti-HBZ cytotoxic T cells into the generated HT-48 mouse model increased survival of these mice suggesting that HBZ might be a candidate for vaccine production (Sugata et al., 2012). Recently, ATL stem cells were identified in an HBZ model (Kuribayashi et al., 2016). In this study, HT-48 cells from *HBZ*-Tg mice were injected intraperitoneally into C57BL/6 mice, then serial transplantation experiments were done to assess the presence of LIC where nine consecutive transplantations were done (Kuribayashi et al., 2016). Surprisingly, HT-48 cells were able to regenerate leukemia in all transplantations. In this model, ATL stem cells were identified as c-kit^+^/CD4^-^/CD8^-^ cells. Compared to the T cell progenitors, reported ATL stem cells had a similar gene expression profile (Kuribayashi et al., 2016).

More recently, a transgenic *HBZ* mouse model using Granzyme B (Gzmb-*HBZ*) was generated (Esser et al., 2017). In addition to splenomegaly, abnormal white cell count, tumors developing after 18 months, in two thirds of the Gzmb-*HBZ* mice, as well as pathologic bone loss and hypercalcaemia were obtained (Kinosada et al., 2017) (**Table 1**).

#### The Double Transgenic HBZ/Tax Mouse Model Fails to Recapitulate ATL

Zhao et al. (2014) established a double transgenic mouse model expressing both Tax and HBZ viral proteins. In this model, both proteins were exclusively expressed in CD4^+^T cells. The concomitant transgenic expression of both Tax and HBZ resulted in skin lesions, T-cell lymphoma, and splenomegaly resembling in part diseases observed in HTLV-I infected individuals (Zhao et al., 2014). In addition, HBZ/Tax double expression resulted in an increase in the number of CD4^+^ memory T cells and Foxp3^+^ T_reg_ cells. Overall, they reported that little difference is seen between the phenotype produced by *HBZ*-Tg model and *HBZ/Tax* double transgenic model. However, in contrast to all published findings, this study reported that “Tax expression alone failed to generate major health problems” and did not result in tumor development (Zhao et al., 2014). In addition, this model resulted in lymphoma and thus failed to generate ATL-like leukemia (**Table 1**).

## Conclusion

In an attempt to unveil the molecular mechanisms dictating ATL development and to advance novel therapeutic options, multiple animal models were utilized. The use of animal models to study HTLV-I infection and ATL development has been instrumental in providing valuable data concerning disease progression and potential therapy targets. Besides ATL patients, mice remain the most valuable and useful tools for *in vivo* investigation of ATL. Generation of ATL xenograft models strikingly advanced the search for targeted therapies for ATL. To assess ATL pathogenesis in a more human setting, humanized models were remarkably helpful. These models are critical for studying early steps of HTLV-I infection. Transgenic ATL models expressing HTLV-I proteins, Tax, HBZ, or both have disclosed the potential roles and contributions of either protein to ATL pathogenesis. In this context, *Tax* transgenic mice, specifically *Lck*-promoter-models, were shown to develop ATL-like leukemia with pathology and molecular changes resembling acute ATL including activation of NF-κB pathway. *Tax* transgenic mice also served as means of testing targeted drug therapies. On the other hand, *HBZ* transgenic mice showed manifestations of systemic inflammation establishing HBZ as a pro-inflammatory protein. Finally, despite the noted progress in disease understanding and its treatment strategies, an animal model that can fully recapitulate the human ATL disease has not been achieved yet.

## Author Contributions

All authors listed have made a substantial, direct and intellectual contribution to the work, and approved it for publication.

## Conflict of Interest Statement

The authors declare that the research was conducted in the absence of any commercial or financial relationships that could be construed as a potential conflict of interest.
